# Sheep and Cattle Are Not Susceptible to Experimental Inoculation with Hazara Orthonairovirus, a Tick-Borne Arbovirus Closely Related to CCHFV

**DOI:** 10.3390/microorganisms8121927

**Published:** 2020-12-04

**Authors:** Julia Hartlaub, Felicitas von Arnim, Christine Fast, Maryna Somova, Ali Mirazimi, Martin H. Groschup, Markus Keller

**Affiliations:** 1 Institute of Novel and Emerging Infectious Diseases, Friedrich-Loeffler-Institut, Suedufer 10, 17489 Greifswald–Insel Riems, Germany; julia.hartlaub@fli.de (J.H.); felicitas.var@mail.de (F.v.A.); christine.fast@fli.de (C.F.); maryna.somova.volodymyrivna@gmail.com (M.S.); markus.keller@fli.de (M.K.); 2 Department of Medicine, Karolinska Institutet, SE-171 77 Stockholm, Sweden; ali.mirazimi@ki.se; 3 National Veterinary Institute, SE-751 89 Uppsala, Sweden

**Keywords:** HAZV, Hazara orthonairovirus, CCHFV, Crimean–Congo hemorrhagic fever, animal model, cross-reactivity

## Abstract

Hazara orthonairovirus (HAZV) is a tick-borne arbovirus closely related to Crimean–Congo hemorrhagic fever orthonairovirus (CCHFV). Whereas CCHFV is a biosafety level (BSL) 4 agent, HAZV is classified as BSL 2, as it is not known to cause any disease in humans. Belonging to the same serogroup as CCHFV, HAZV might act as a model which can provide a better understanding of this important zoonosis. Furthermore, the serological relatedness may cause diagnostic problems if antibodies against HAZV interfere with current CCHFV serological assays. Therefore, sheep and cattle—important natural hosts for CCHFV—were experimentally infected with HAZV to prove their susceptibility and evaluate potential antibody cross-reactivities. According to this study, neither sheep nor cattle are susceptible to experimental HAZV infections. Consequently, the HAZV infection in ruminants is clearly distinct from CCHFV infections. Sera of immunized animals weakly cross-reacted between HAZV and CCHFV in immunofluorescence and immunoblot assays, but not in commercial CCHFV ELISAs commonly used for field studies.

## 1. Introduction

The genus *Orthonairovirus* within the order *Bunyavirales* comprises 43 predominantly tick-borne arboviruses including several viruses associated with severe human and livestock diseases [1,2].

The genome of these negative-sense single-stranded RNA viruses is divided into three segments: the large (L) segment encodes for the viral RNA-dependent RNA-polymerase and the medium (M) segment for a precursor protein, which is cleaved into the glycoproteins Gn and Gc and an additional non-structural protein (Nsm). The small (S) segment encodes for the nucleocapsid protein (N) [2,3].

*Orthonairoviruses* are classified into 15 species and are currently assembled into different serogroups according to their antigenic relationships based on hemagglutination inhibition (HI), infectivity neutralization, complement fixation (CF) and immunofluorescence (IF) tests [3,4,5].

The most momentous genus member is Crimean–Congo Hemorrhagic Fever Virus (CCHFV) causing severe hemorrhagic fever and fatalities rates from 5–30% in humans [6]. Due to its high pathogenicity, laboratory work is limited to high containment level (BSL 4). Distribution is correlated to the presence of its main tick vector *Hyalomma* ssp., but in consequence to global warming, the area where competent ticks are endemic is enlarging. CCHFV is already widespread in Africa, Asia, the Middle East and south-eastern Europe with the potential to emerge to further regions [7,8].

Belonging to the same serogroup as CCHFV, Hazara orthonairovirus (HAZV) is so far not known to cause disease in either humans or in animals, except in experimentally infected animals such as knockout mice [9], and is therefore classified as BSL 2. The prototype strain JC280 was isolated in 1964 from a pool of six *Ixodes redikorzevi* ticks collected from the vole *Alticola roylei* at an elevation of 12,000 ft in subarctic terrain in the Kaghan valley, Hazara District in West Pakistan [10,11]. There have not been any further isolations of HAZV from ticks, animals or humans limiting research to one single virus strain. A serological study conducted in Pakistan detected antibodies at low titers in a jird (*M. hurrianae*) without detectable antibodies for CCHFV [12]. In India, 643 human and 655 animal sera were screened for HAZV antibodies, but without positive results [13]. There is only little serological evidence that humans are susceptible to HAZV infection: 4 out of 150 human sera collected in Hazara District, Karachi, Lahore and Dacca showed positive reactions in HI and CF assays [14].

HAZV shares many structural, biochemical and cellular properties with CCHFV. Comparison of the complete sequence of the S segment and partial genome sequences (442 bp) of the L segment revealed 60 and 70% nucleotide sequence identity between HAZV and different CCHFV strains, respectively [15,16].

Several studies have been performed proposing HAZV as a model for nairoviral infections. Research focuses not only on in vitro work concerning cellular and molecular mechanisms [17,18,19,20,21], but also on the establishment of suitable in vivo models, which could contribute, e.g., to drug or vaccine development. Several laboratory animals have already been experimentally infected with HAZV in order to characterize the virus and to elucidate its possible role as a model virus for CCHFV. HAZV induced lethal infection of embryonated chicken eggs [22] as well as lethal infection of B6-IFNAR^tmAgt^ mice (type-I interferon receptor knockout), which showed the same clinical signs and pathology such as those reported in previous trials with CCHFV [9].

This study focuses on common hosts of *Orthonairoviruses*: ruminants play an important role in the transmission cycle of CCHFV even though they do not show clinical signs [8]. Ticks can acquire the virus while blood-feeding on viremic animals and may further transmit the virus to humans. Humans can also be infected directly when they come into contact with blood, body fluids or tissues of viremic animals [6].

Due to their close relationship, HAZV might act as a surrogate for CCHFV infections in ruminants and can contribute to a better understanding of nairoviral infections in this species. The aim of this study is to investigate whether sheep and cattle are susceptible to HAZV infection. Research concerning the pathogenesis, virus–host interactions and immune responses in ruminants could be easily performed under BSL 2 conditions, while work with CCHFV is limited to a few facilities, which can conduct large animal trials under BSL 4 conditions.

Furthermore, many serological monitoring programs worldwide focus on the prevalence of CCHFV antibodies in ruminants to determine the current distribution of the virus and consequently evaluate the risk for human infection and disease [23,24]. HAZV and CCHFV belong to the same serogroup and therefore it cannot be excluded that serological assays are not able to distinguish between antibody responses following these infections. CCHFV is endemic in Pakistan [25], underlining the need for discriminative serological assays for the detection of HAZV and CCHFV antibodies. As the real distribution of HAZV still remains undetermined, it could be possible that animals (and humans) infected with non-pathogenic HAZV might produce unrecognized false positive results in CCHFV serological assays. Therefore, the second aim of this study is to re-evaluate the impact of occurring cross-reactivities between HAZV and CCHFV, as studies performed earlier claimed significant cross-reactivities [4,14,26].

## 2. Materials and Methods

### 2.1. Virus and Cells

HAZV Strain JC 280 as well as SW13 cells were kindly provided by Ali Mirazimi, Karolinska Institutet, Stockholm, Sweden. For indirect immunofluorescence assays, Vero E6 cells (Collection of Cell Lines in Veterinary Medicine, Friedrich-Loeffler-Institut, Greifswald, Germany) were employed. SW13 cells were cultivated in L-15 Medium (Sigma Aldrich, Darmstadt, Germany) supplemented with 5% fetal calf serum (FCS), whereas Vero E6 cells were cultivated in Modified Eagles Medium (MEM, Collection of Cell Lines in Veterinary Medicine, Friedrich-Loeffler-Institut, Greifswald, Germany) supplemented with 10% FCS. Virus stocks were grown in SW13 cells. Growth kinetics were performed revealing maximum viral titers 48 h post-infection (up to 10^7.8^ TCID_50_/mL). A cytopathic effect becomes first visible 3 days post-infection (dpi).

### 2.2. Quantification

The virus titer was determined using two different methods depending on the intended use of each virus stock. All titrations were performed at least in triplicate.

TCID_50_ titer: SW13 cells were seeded in 96-well plates and incubated for approx. 24 h at 37 °C, 5% CO_2_ before medium was removed and 100 µL of 10-fold serial diluted HAZV was added to the monolayers. Cells were incubated for 1 h before 100 µL of maintenance medium L-15 containing 2% FCS and supplemented with 1 µL/mL penicillin and 0.5 µL/mL streptomycin were added. Plates were incubated for 6 days and afterwards fixed with neutral buffered formalin and stained with 1% crystal violet. Cytopathic effect (CPE) was evaluated for each well. The 50% Tissue Culture Infective Dose (TCID_50_) was calculated as described by Spearman and Kaerber.

Plaque forming unit (pfu) titer: for the plaque assay, SW13 cells were grown in 6-well plates. Inocula of serial 10-fold dilutions of the virus stock were prepared and incubated for 1 h at 37 °C, 5% CO_2_ before removing the medium of the 6-well plates and adding 500 µL of the virus dilutions in duplicate. This modification was chosen to receive comparable plaque counts such as in the protocol for the plaque reduction neutralization test (PRNT) where sera and virus dilutions were incubated for 1 h for the neutralization process. Cells were then incubated for 1 h at 37 °C, 5% CO_2_ and gently rocked every 15 min. Later, inocula were removed and cells were overlaid with 1.8% Bacto-Agar (mixed 1:1 with MEM, 4% heat-inactivated FCS, 2 µL/mL penicillin and 1 µL/mL streptomycin). A second overlay consisting of 0.1% crystal violet and neutral buffered formalin was added after 4 days. After a further 24 h, overlays were removed. Plaques were counted visually and titers calculated and expressed as pfu/mL.

When comparing TCID_50_ and pfu titers of the same virus stock, the TCID_50_ titers were around 1 log scale higher than the pfu titers.

### 2.3. Quantitative Reverse Transcriptase Polymerase Chain Reaction (RT-qPCR)

To determine the load of viral genome equivalents in samples suspected to be positive for HAZV, a target region in the polymerase gene located on the L segment of the viral genome was selected. The used primer and probe are listed in Table 1.

RT-qPCR was performed using the QuantiTect Probe RT-PCR Kit (Qiagen, Hilden, Germany) in a total reaction volume of 25 μL according to the manufacturers’ instructions. The reaction mix comprises a 12.5 µL 2× reaction master mix, 0.25 µL RT-Mix, 10 pmol of each HAZV primer and 1 pmol of HAZV probe. Water was used to fill up to a volume of 20 µL and finally 5 μL of template RNA was added. As internal extraction control, MS2 bacteriophage RNA was added to the samples before RNA isolation. Specific MS2 primer/probe mix was used for detection [27]. A synthetic RNA comprising the target region of the RT-qPCR was utilized as calibrator for quantification and as a positive control for every RT-qPCR plate. The synthetic calibrator was generated by in vitro transcription from the corresponding DNA-sequence, which contains at the 5′-end an additional T7 promotor sequence for in vitro transcription.

The real-time RT-qPCR was performed with a CFX96 Real-Time PCR Detection System (Bio-Rad Laboratories, Hercules, CA, USA). The cycling conditions used were as follows: 50 °C for 30 min (reverse transcription), 95 °C for 15 min (reverse transcriptase inactivation/Taq polymerase activation), followed by 35 cycles at 95 °C for 10 s (denaturation), 60 °C for 30 s (annealing) and 72 °C for 25 s (elongation). Fluorescence data were collected after each 60 °C step and analysis of the fluorescence data was conducted with the CFX Manager software (Bio-Rad Laboratories, Hercules, CA, USA).

### 2.4. Recombinant Expression of HAZV N Protein

The coding region for the N protein of the Hazara virus strain JC280 (S segment; genbank accession number KC344857) was amplified by RT-PCR using the forward primer HAZVS74F 5′-CTAGCGACATATGTCAGCGAGAATGGAGAACAAGAT-3′ and the reverse primer HAZVS1676R 5′-GCTCTAGACTCAAAGATATCGTTGCCGCAC-3′. In addition to the homologous sequences, the used primer included restriction sites NdeI and XbaI, respectively (underlined). After amplification, the PCR product was cloned into the vector pCR2.1 (TA Cloning Kit, invitrogen, Waltham, MA, USA). Subsequently, the coding region was cloned into the prokaryotic expression vector pET19b using the restriction enzymes NdeI and XbaI. Sequence identity and correct insertion was verified by sequencing.

For recombinant expression, the *E. coli* K12 strain BL21(DE3) was transformed with the pET19b-HAZV-N plasmid. Expression was induced by addition of Isopropyl-β-D-thiogalactopyranosid (IPTG, MP Biomedicals, Irvine, CA, USA) during the logarithmic growth phase of the bacteria. After 4 h of expression, bacteria were sedimented and protein isolation and purification were carried out using the N-terminal His-Tag. For the purification step according to the Expressionist protocol by Qiagen, Ni-NTA Sepharose (Qiagen, Hilden, Germany) was used.

### 2.5. Serology

#### 2.5.1. Development of an Indirect IgG-ELISA Based on Recombinant Antigen

Bovine protocol: purified HAZV N protein was diluted in phosphate-buffered saline (PBS) containing 0.5% BSA (Albumin fraction V, Merck, Darmstadt) to a final concentration of 2 µg/mL and 100 µL per well was coated on Greiner F plates for 1 h at 37 °C. Four wells were prepared for each sample: two with the recombinant protein and two with the antigen dilution buffer only. Plates were washed three times with 200 µL/well wash solution (PBS containing 0.05% Tween20). In total, 200 µL of blocking buffer (IDVet, Grabels, France) was added and plates were incubated for 1 h at room temperature. Plates were pushed on cellulose for drying. Sera were diluted 1/20 in IDVet buffer No. 3 and 100 µL/well was added. The following incubation steps were all performed for 1 h at 37 °C. Plates were washed three times with 200 µL/well wash solution and the conjugate (Peroxidase-conjugated AffiniPure Goat Anti-Bovine IgG, Jackson ImmunoResearch, Cambridgeshire, UK) was diluted 1/10,000 in IDVet Buffer No. 11 and 100 µL was added to each well. Again, plates were incubated and washed 3 times afterwards with 200 µL/well with wash solution. In total, 100 µL of the substrate (1-Step^TM^ Ultra TMB-ELISA, Thermo Scientific, Braunschweig, Germany) was added to each well and incubated for approx. 10 min in the dark. The reaction was stopped with 100 µL/well 1M H_2_SO_4_. Optical density was measured at 450 nm and the corrected OD values were calculated as follows: mean OD_450_ with antigen-mean OD_450_ without antigen. Afterwards, the percentage of the corrected OD value of the sample in comparison to the positive control (immunized cattle, HAZV-IMMU-calf-1) was calculated. Negative cut-off values were determined employing 100 sera from German cattle.

Ovine protocol: only differences between the ovine and bovine protocol are mentioned. Antigen was diluted in PBS containing 1% albumin from eggs (VWR Chemicals, Darmstadt, Germany) and plates were coated overnight at 4 °C. All washing steps were performed with PBS containing 1% Tween20. The secondary antibody (Peroxidase-conjugated AffiniPure Donkey Anti-Sheep IgG, Jackson ImmunoResearch, Cambridgeshire, UK) was diluted to 1/3000. An immunized sheep (HAZV-IMMU-sheep-1) served as the positive control.

#### 2.5.2. Indirect Immunofluorescence Assay

Vero E6 cells were seeded in 96-well plates 24 h before infection. HAZV was diluted in modified Eagles Medium (MEM) supplemented with 5% FCS, 1 µL/mL Penicillin and 0.5 µL/mL Streptomycin to a multiplicity of infection (MOI) of 0.5. Medium was removed and 100 µL/well virus dilution was added to the odd rows, whereas the even rows served as non-infected controls and medium without HAZV was added to exclude non-specific reactions of the sera to the cells or buffers used. Plates were incubated for 24 h at 37 °C, 5% CO_2_ and then fixed with methanol–acetone (1:1 ratio) and stored at −20 °C until used.

For the indirect immunofluorescence assay (iIFA), plates were thawed and washed 3 times with 200 µL/well PBS. The blocking step consisted of 1 h blocking at room temperature with 200 µL/well PBS containing 5% goat serum (for testing bovine sera) or 5% fetal calf serum (for testing sheep sera). Test sera were diluted to 1/50 in PBS and 100 µL was added to one HAZV infected well and one non-infected control well. Plates were incubated for 1 h at 37 °C and washed with 3 × 200 µL PBS. The bovine conjugate (Alexa Fluor^®^ 488 conjugated AffiniPure Goat Anti-Bovine IgG, Jackson ImmunoResearch, Cambridgeshire, UK) was diluted to 1/400 in PBS and 50 µL was added to each well. The sheep conjugate (Donkey anti-Sheep IgG (H&L), DyLight^®^ 488 Conjugate, ImmunoReagents, Raleigh, NC, USA) was diluted to 1/200 in PBS. After 1 h at 37 °C, plates were again washed 3 times with 200 µL/well. Counterstaining of the nucleus was performed using DAPI (Sigma Aldrich, Darmstadt, Germany) diluted to 1/5000 in PBS for 10 min.

#### 2.5.3. Plaque Reduction Neutralization Assay (PRNT)

Sera were heat-inactivated for 30 min at 56 °C and serially diluted in maintenance medium starting with a 1/2 ratio. Virus stocks were diluted to a final concentration of 200 pfu/mL and 600 µL of the serum dilution was added to the same volume of virus dilution and mixed gently. Inocula were incubated for 1 h at 37 °C with 5% CO_2_ and afterwards 500 µL was added in duplicate to the SW13 6-well plates. One plate was used for back-titrations of the virus stock (reconfirming the actual pfu titer of the used virus stock) and one additional plate was prepared containing the 1/2 diluted working dilution of the virus stock. The rest of the protocol was equivalent to the plaque assay presented before. Plaques were counted and the mean of the control plate was determined and set as 100%. For each sample, the mean plaque count for every dilution was calculated and a serum dilution was declared positive if 80% of plaques were reduced (PRNT_80_).

#### 2.5.4. CCHFV Diagnostic Assays

The multispecies double antigen ELISA (IDVet, Grabels, France) was applied according to the manufacturer’s instructions. An in-house ELISA based on recombinant N protein and a species-adapted protocol for the commercial human Vector-Best IgG ELISA (Vector-Best, Novosibirsk, Russia) and an immunofluorescence assay (Euroimmun, Luebeck, Germany) were used as described before [28].

Western blot analysis was conducted according to standard procedures. For the immune detection, sera were diluted 1/20 in 5% skim milk and incubated for 1 h. Membranes were washed with PBS-Tween20 and the conjugate (Peroxidase-conjugated AffiniPure Goat Anti-Bovine IgG or Peroxidase-conjugated AffiniPure Donkey Anti-Sheep IgG, Jackson ImmunoResearch, Cambridgeshire, UK) was diluted to 1/3000 in 5% skim milk and added to the membrane. After 1 h, membranes were washed again and the substrate (SuperSignal West Pico PLUS Chemiluminescent Substrate, Thermo Scientific, Braunschweig, Germany) was applied. Chemiluminescence was measured with ChemiDoc^TM^ Touch Imaging System (Bio-Rad Laboratories, Hercules, CA, USA) and blots were analyzed with Image Lab software (Bio-Rad Laboratories, Hercules, CA, USA).

### 2.6. Immunization Studies

One calf (HAZV-IMMU-calf-1) was immunized with formalin-inactivated HAZV: 3 boosts, dose: 4.6 × 10^6^ TCID_50_ HAZV, adjuvant: TS6 (Merial, Toulouse, France). One sheep (HAZV-IMMU-sheep-1) was immunized equally: 3 boosts, dose: 1.2 × 10^9.4^ TCID_50_, adjuvant: GERBU Adjuvant P (GERBU Biotechnik GmbH, Heidelberg, Germany). Each animal was injected at four different locations (2x breast, 2x neck) with 2.5 mL per injection site.

### 2.7. HAZV Challenge Studies

Animal trials were conducted under BSL 2 conditions. Experiments were performed in compliance with national and European legislations, and were approved by the competent authority of the Federal State of Mecklenburg-Western Pomerania, Germany (LALLF 7221.3-1-011/19, approval date: 07.05.2019).

Twelve sheep (German black-headed mutton, 6 female/6 male, age 3–4 months, obtained from Friedrich Loeffler Institut, Mariensee, internal animal codes: A1-6, B1-6) were inoculated subcutaneously in the lateral chest wall with 10^6^ TCID_50_ HAZV (injected volume: 3 mL). As mock controls, two sheep (C1, C2) were kept separately and inoculated with cell culture medium only. Every day clinical scores (considering behavior, breathing rate, food uptake and injuries) were determined and rectal body temperature was measured. Blood (whole blood and serum) and swab (nasal and rectal) samples were collected prior to infection and at different time points post-infection. (1st week every day alternating half of the sheep, 2nd–4th week: every 2nd day sampling of half of the sheep). Necropsies were performed 4 dpi (A1, A2, B1, B2) when the approx. start of viremia was expected and 10 dpi when RT-qPCR positive organ samples were expected to reveal the organ tropism of HAZV (A3, A4, B3, B4, C1). The last 4 animals (A5, A6, B5, B6) + 1 mock control (C2) were kept for a further 18 days in order to obtain seropositive serum samples.

Four calves (Frisian Holstein, 2 female/2 male, age 3–5 months, obtained from commercial suppliers: RinderAllianz GmbH, internal animal codes: D5, D6, E5, E6) were infected subcutaneously in the lateral chest wall with 10^6^ TCID_50_ HAZV (injected volume: 3 mL), respectively. One calf (F2) was inoculated with cell culture medium only and was kept in a different stable as a mock control. Procedures were described for the sheep, but all the animals were kept for 28 days.

Daily sampling of the animals: blood was centrifuged and serum was separated, swab samples were shaken for 30 min in 1 mL of L-15 medium and the fluid was then transferred to a new falcon. These samples as well as EDTA blood were stored at −70 °C prior to RNA isolation.

Necropsies: animals were euthanized with Pentobarbital intravenously (Release^®^, WDT, Garbsen, Germany) and necropsied. A diverse panel of tissue samples was collected (injection site (lateral chest wall), Ln. cervicalis cran., heart muscle, lung, small intestine, large intestine, rectum, liver, kidney, spleen, Lnn. iliaci med., Lnn. retropharyngeales med., tonsils, conchiae, conjunctivae and brain).

Tissue samples were homogenized in 1 mL of L-15 medium using a TissueLyzer (Qiagen, Hilden, Germany). Total RNA from the organ homogenates, serum and whole-blood samples was extracted using the King Fisher 96 Flex purification system (Thermo Scientific, Braunschweig, Germany) in combination with the NucleoMag Vet Kit (Macherey-Nagel, Düren, Germany) according to the manufacturers’ instructions. As an internal extraction control, an MS2 bacteriophage was added prior to RNA extraction to each sample in order to avoid false negative results in the following HAZV-RT-qPCR.

## 3. Results

### 3.1. Sheep and Bovine Challenge Study with HAZV: Clinical Signs and Gross Pathology

Twelve sheep were inoculated subcutaneously with 10^6^ TCID_50_ HAZV and clinical scores were determined over the next 28 days. Moreover, blood and swab samples were taken regularly. Necropsies of groups of four sheep, respectively, were carried out at 4 dpi (suspected start of viremia) and 10 dpi (to reveal the suspected organ tropism of HAZV), and after another 18 days (to obtain seropositive serum samples). Likewise infected, clinically assessed and sampled were four calves, but all animals were kept for 28 days.

Virus back-titrations (standard titrations reconfirming the actual virus titer) of the injected inocula for the sheep and calves contained the expected amount of the virus, respectively.

**Figure 1:**
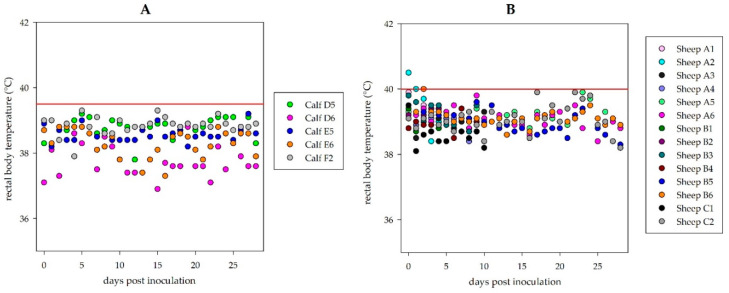
Rectal body temperature during the observation period: No fever could be detected in inoculated cattle (**A**) or sheep (**B**). Each animal is represented by a single pattern. Physiological rectal body temperatures range up to 39.5 °C for the calves and up to 40.0 °C for the sheep, respectively (illustrated by the red line in each graph); short term higher temperatures may be animal handling/excitement artefacts.

During the observation period of 28 days, neither the sheep nor the calves showed any clinical signs. No rise of the rectal body temperature was registered (Figure 1). Moreover, material from euthanized and necropsied animals did not show any lesions related to viral disease in gross pathology.

All swab (rectal and nasal), whole-blood, serum and tissue samples were analyzed by a HAZV specific RT-qPCR. None of the samples gave a positive signal, whereas the positive results for the extraction controls (MS2) verified that the RNA isolation and RT-qPCR functionality were successful. Consequently, inoculated animals did not develop viremia and did not shed the virus. No virus load was revealed in any tissue including the injection site and tributary lymph nodes.

### 3.2. Establishment of HAZV N Protein ELISAs

**Figure 2:**
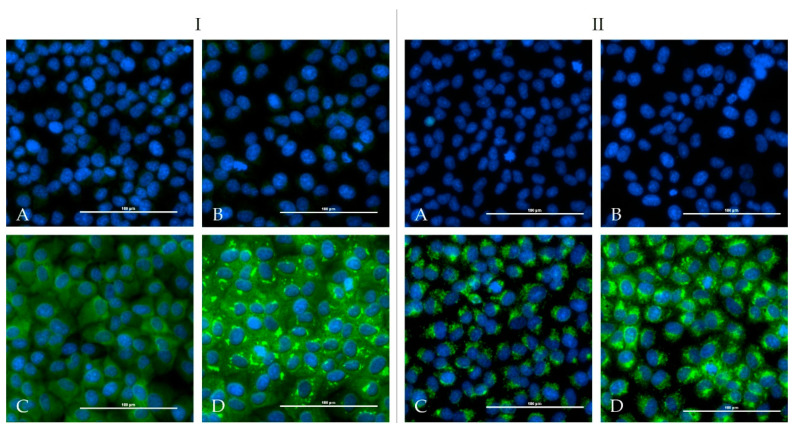
Indirect immunofluorescence assay with HAZV infected Vero E6 cells: serum prior immunization did not lead to positive results in non-infected controls (**A**) or infected cells (**B**). Post-immunization serum (1/50 diluted) lead to a specific fluorescence signal in infected cells (**D**) whereas non-infected controls (**C**) did not show specific staining patterns and are therefore evaluated as negative. Panel **I** shows the immunized calf (HAZV-IMMU-calf-1) and Panel **II** the immunized sheep (HAZV-IMMU-sheep-1) results.

One calf and one sheep were immunized repeatedly with an adjuvated-inactivated virus vaccine via subcutaneous injection. Serum of the immunized calf (HAZV-IMMU-calf-1) showed a highly virus specific staining on HAZV infected cells in an indirect immunofluorescence assay. Serum of the immunized sheep (HAZV-IMMU-sheep-1) also led to staining of the HAZV infected cells, but non-specific background signals were higher as compared to pre-vaccination sera. However, when directly comparing infected vs. non-infected wells, the post-immunization serum was clearly positive (Figure 2).

Sera from these animals also reacted with HAZV N protein coated onto ELISA plates. For optimized indirect HAZV N protein ELISA protocols, a variety of different serum and secondary antibody concentrations were eventually tested to find optimal signal/background noise ratios. The reactivities of these sera were eventually set as 100% (positive reference) and field sera from sheep and bovines were run to determine cut-off values.

For this purpose, 100 sera from German sheep were run in the indirect HAZV ELISA and the obtained results were used for cut-off definition (mean + 3 × standard deviation = 35.8%). With this cut-off, the diagnostic specificity was 98%. Moreover, 100 sera from German cattle were tested with the indirect HAZV ELISA and the cut-off was determined at 18.5%. According to this cut-off value, the diagnostic specificity was 98%. The diagnostic sensitivity of both assays could not be determined as just one single seropositive sample from each species was available.

### 3.3. Serological Results for the Challenge Animals

**Figure 3:**
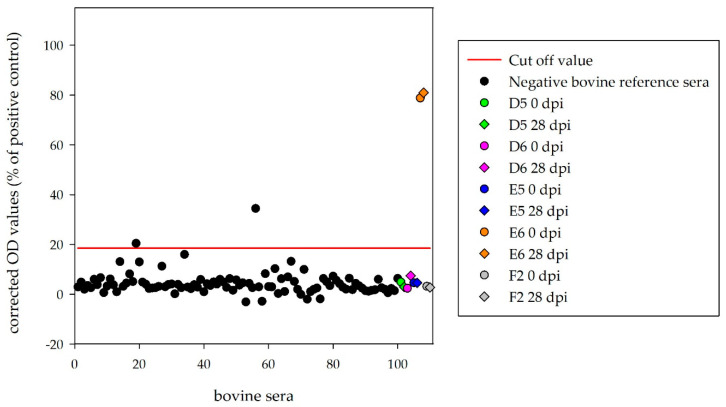
Indirect bovine HAZV N protein ELISA: corrected OD values for the 100 negative reference sera from German cattle demonstrate 98% specificity of this ELISA. OD values were below the determined cut-off for D5, D6, E5 and F2 prior and post-inoculation. Calf E6 tested positive prior inoculation.

Sera of the four inoculated calves (D5, D6, E5, E6) and the mock control (F2) were tested with the HAZV N protein ELISA. Corrected OD values (28 dpi) were clearly below the cut-off (18.5%) for D5 (2.9%), D6 (7.5%), E5 (4.6%) and F2 (2.7%). Surprisingly, one infected calf (E6) tested as strongly positive (81.0%), with equivalent results for the serum collected before HAZV inoculation (78.8%) (Figure 3).

**Figure 4:**
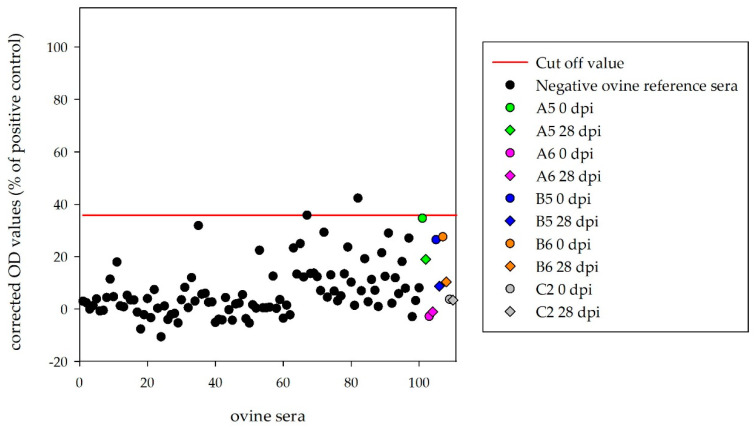
Indirect ovine HAZV N protein ELISA: corrected OD values for the 100 negative reference sera from German sheep demonstrate 98% specificity of this ELISA. OD values were below the determined cut-off for all HAZV inoculated sheep.

Sera of the four inoculated sheep, which were kept until the end of the experiment (A5, A6, B5, B6), and the mock-control (C2) were tested with the ovine HAZV N protein ELISA. Corrected OD values (28 dpi) were below the determined cut-off (35.8%) for A5 (19.0%), A6 (−1.1%), B5 (8.7%), B6 (10.3%) and C2 (3.4%) (Figure 4).

**Figure 5:**
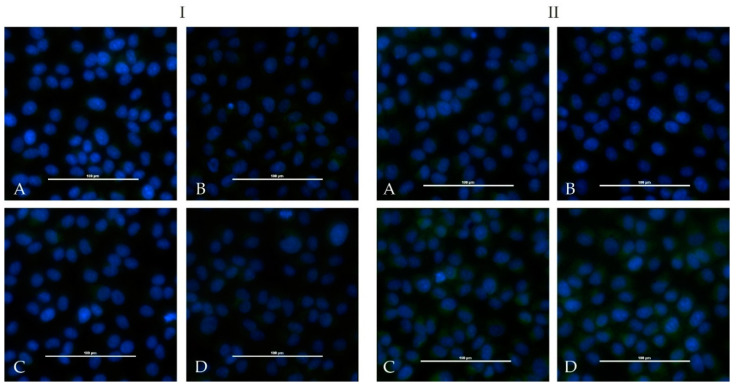
Indirect immunofluorescence assay with HAZV infected Vero E6 cells: no differences between wells with serum prior infection ((**A**): non-infected controls, (**B**): infected wells) and wells with serum 28 dpi ((**C**): non-infected controls, (**D**): infected wells) were registered in infected calves ((**I**), example calf E5)) and sheep ((**II**), example sheep A5)).

None of the sera from the challenged four calves and from the four sheep showed positive iIFA reactions for HAZV (Figure 5). Sera of the immunized calf (HAZV-IMMU-calf-1) and sheep (HAZV-IMMU-sheep-1) were run in parallel as positive controls (depicted in Figure 2).

Weak levels of neutralizing antibodies were detected by PRNT in the serum of the immunized calf (HAZV-IMMU-calf-1), leading to 80% reduction in plaques until the 1/8 dilution (PRNT_80_: 1/8). Sera of the immunized sheep as well as of the challenged calves and sheep did not show any neutralizing activity.

### 3.4. Assessment of Serological Cross-Reactions of CCHFV and HAZV Induced Antibodies

**Figure 6:**
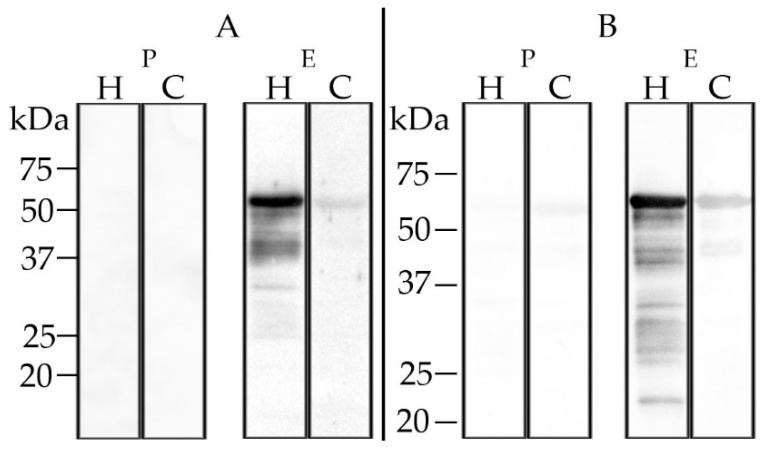
Western blot analysis with recombinant HAZV (H) and CCHFV (C) N protein: HAZV antiserum reacted strongly with HAZV N protein and showed weak cross-reaction with CCHFV N protein when tested with calf (HAZV-IMMU-calf-1, panel (**A**)) and sheep (HAZV-IMMU-sheep-1, panel (**B**)) post-immunization sera (E). Pre-immunization sera (P) did not lead to positive results.

The calf hyperimmune serum gave no positive results in the in-house CCHFV ELISA, the Vector-Best ELISA or in the double antigen ELISA. The sheep hyperimmune serum tested positive with the Vector-Best ELISA, but not with the other ELISAs (data not shown). Western blot analysis employing recombinant CCHFV N protein revealed very weak cross-reactions (Figure 6). Blood before the immunizations did not test positive in any of these assays.

**Figure 7:**
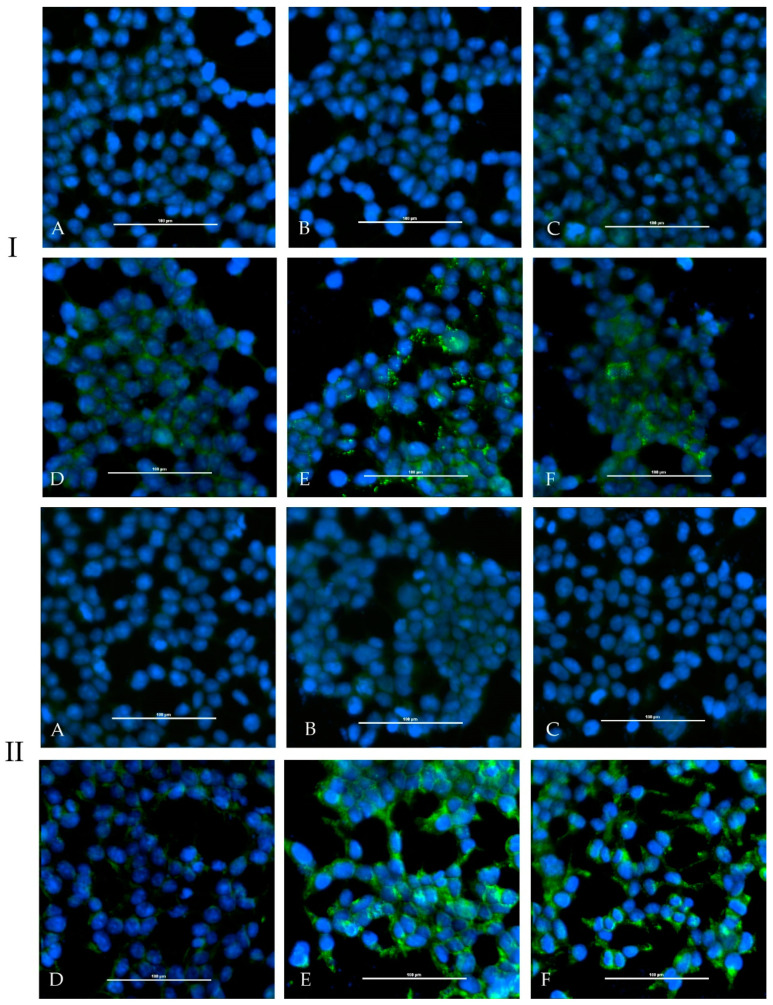
Indirect immunofluorescence assay (Euroimmun, Luebeck) A+D (cell control), B+E (CCHFV GPC, glycoprotein C), C+F (CCHFV N): Sera prior immunization (**A**–**C**) and post-immunization (**D**–**F**) are depicted for the immunized calf (HAZV-IMMU-calf-1, panel **I**) and sheep (HAZV-IMMU-sheep-1, panel **II**). A specific fluorescence signal could be detected for both post-immunization sera on cells transfected with CCHFV GPC and CCHFV N, whereas sera prior immunization tested negative.

In the adapted iIFA (Euroimmun), the sera showed weak fluorescence signals on transfected cells with CCHFV N protein as well as CCHFV glycoprotein C (Figure 7).

Results concerning the evaluation of cross-reactivities between HAZV and CCHFV are summarized in Table 2.

## 4. Discussion

In this study, ruminants were challenged with HAZV, as they are hosts and reservoirs for the closely related viruses Crimean–Congo hemorrhagic fever orthonairovirus (CCHFV), Dugbe orthonairovirus (DUGV) [29] and Nairobi sheep disease orthonairovirus (NSDV) [30]. According to the data presented in this report, sheep and cattle are not susceptible to experimental challenges with Hazara orthonairovirus, as they showed neither clinical signs nor developed viremia nor virus specific antibodies. This is well in line with anecdotal evidence, as no natural infections of ruminants with HAZV have ever been reported. However, prevalence data on HAZV are rather scarce. Even in Pakistan, where HAZV was first isolated [10], no larger seroprevalence study for HAZV specific antibodies in ruminants has ever been conducted.

Several reasons can lead to the lacking susceptibility of sheep and bovines. Tick-borne HAZV may generally not be virulent for these species or for mammalians in general. There is only one published animal challenge study involving multiple mammalian species, which was carried out in the late 1970s where various CCHF viruses were used [31], amongst which HAZV was also mentioned. However, data documentation in this report is more than fragmentary and imprecise—i.e., there is neither comprehensive and complete information on the experimental setup nor on the results. However, HAZV was also thought to cause mild clinical infections in several experimentally infected animal species (mice, rats, guinea pigs, rabbits, hamsters, rhesus monkeys, donkeys, sheep and calves). It remains unclear which route of inoculation or viral dose was chosen and if really all of the listed animals developed a viremia and showed seroconversion after inoculation of every member of the CCHF group virus strains. Comparing the methods used for the detection of viremia (this study: HAZV specific RT-qPCR vs. Smirnova: suckling mice inoculation) and seroconversion (this study: ELISA based on recombinant antigen, iIFA, PRNT vs. Smirnova: complement fixation test), we claim that the assays utilized in this present study are more specific.

Another factor modulating a challenge study outcome may be the infection mode that is used. We have used the subcutaneous needle injection to mimic the tick bite HAZV transmission route which may still be suboptimal. There might be differences in the amount of virus transmitted by a tick bite in comparison to the inoculum used in this study. Furthermore, the role of the tick saliva has to be taken into consideration, as it may contain additional factors modulating the local immune response and/or enhancing virus infectivity (so-called saliva-assisted transmission SAT) [32]. In a recent study investigating the HAZV vector competence of *Ixodes ricinus* ticks, B6-IFNAR^tmAgt^ mice were both infected via an in vivo tick feeding system and intradermal injection. Although it was assumed that the virus titer applied was equivalent, mice infected intradermally died more rapidly whereas mice infected by tick bites did not show clinical signs, hence indicating the absence of a SAT effect. One reason might be the suppression of migration of macrophages and dendritic cells leading to a delayed and diminished manifestation of the disease [33].

Another reason for the failure of HAZV to infect ruminants might be the putatively high cell culture passage of the virus stock. However, next generation sequencing (NGS) of the complete HAZV genome used in our study revealed only a single silent mutation in the M segment compared to the GenBank HAZV reference sequences (KP406723, KP406724, KP406725) (data not shown).

There are hardly any diagnostic tests available at present to measure immune responses following HAZV infections. We have therefore established a recombinant N protein based on indirect ELISA using reference sera from an immunized sheep and a calf as well as panels of 100 sheep and 100 cattle sera from Germany, all of which served as negative controls. In both immunized animals, seroconversion was confirmed by de novo reactivity in the indirect ELISA and by indirect immunofluorescence staining on HAZV infected Vero E6 cells. The calf serum also tested as low positive (PRNT_80_ = 1:8) in the PRNT. Nairoviruses are not known to induce high levels of neutralizing antibodies [34].

One calf (E6) tested positive in the HAZV ELISA even before the HAZV challenge. As the calf serum tested negative with other assays (iIFA, PRNT, Western blot), the ELISA result is considered to be non-specific. Additionally, this serum also tested positive in the Friedrich-Loeffler-Institut (FLI) in-house CCHFV- and DUGV-indirect ELISAs, but did not cross-react with recombinant N proteins of more distantly related *Bunyaviruses,* such as Rift Valley fever phlebovirus or Bunyamwera orthobunyavirus, even though these proteins were expressed and purified following equivalent protocols. Future research is needed to determine whether cross-reactivities to other nairoviral N proteins can be found more frequently in Germany and elsewhere.

To investigate cross-reactivities between HAZV and CCHFV, the sera of the immunized animals were tested in the currently available CCHFV diagnostic tests. The sera were tested in three different CCHFV ELISAs: The in-house CCHFV ELISA is based on recombinant N protein of a strain of clade V (Kosovo), the antigen of the Vector-Best ELISA consists of inactivated whole-virus of a strain of clade IV (Uzbekistan) and the IDVet double antigen ELISA uses recombinant N protein of a CCHFV strain from clade III (Nigeria). The calf serum did not test positive in any of these CCHFV ELISAs, although it tested positive in all of the developed HAZV serological tests—hence, we assume that these CCHFV assays can indeed discriminate between antibodies against HAZV and CCHFV.

The pre- and post-vaccination sheep serum tested negative in the commercial double antigen ELISA and in a modified version of the published inhouse ELISA [23], in which bovine serum albumin was replaced by ovine serum albumin in the coating buffer. This modification was chosen, as post-vaccination serum reacted with the actual coating buffer (without CCHFV antigen). Similar non-specific background reactions to the blocking compounds were also seen for the Vector-Best ELISA. This indicates that the antibodies themselves did not cross-react with CCHFV. For both immunized animals, weak positive signals were observed in the species-adapted iIFA and on immunoblotted recombinant CCHFV N protein (clade III), indicating a lack of specificity of those assays.

In summary, ELISAs were clearly able to discriminate between HAZV and CCHFV antibodies, while cross-reactivities between these viruses in iIFA and WB may occur. However, it should be possible to neglect these even in CCHFV field studies, as sheep and cattle are obviously not highly susceptible. Taking into consideration that the Nairobi sheep disease serogroup is genetically and antigenetically very closely related to the CCHFV serogroup [35], viruses from this serogroup might also interfere with CCHFV diagnostics. Serological relationships have already been studied in the past [36] but a lot of contradictory results highlight the importance of re-evaluation of these cross-reactivities applying up-to-date techniques. Therefore, equivalent to HAZV, animal challenge and assay development studies are underway currently in our group in order to elucidate the DUGV and NSDV (both BSL 3) pathogeneses and immune response in ruminants.

## Figures and Tables

**Table 1:** Sequences of primer and probes used for the detection of Hazara orthonairovirus (HAZV) genome.

**Primer/Probe** **HAZV L Segment**	**Sequence 5′→3′**	**Localization According to Reference Sequence (GenBank Accession Number: KP406723)**
HAZVL11737F	TCCCAGGGCCGAGTCTGAAGA	11,737–11,757
HAZVL11969R	TCGTTCCCCCACACCCCAATTT	11,969–11,948
HAVZL11795FAM	FAM-TGGGACAGTGACTGACGGTGTGGA-BHQ1	11,795–11,818
**Primer/Probe** **Internal Extraction Control**	**Sequence 5′→ 3′**	**Localization According to Reference Sequence (GenBank Accession Number: MK213795)**
MS2F	CTCTGAGAGCGGCTCTATTGGT	2233–2254
MS2R	GTTCCCTACAACG AGCCTAAATTC	2333–2310
MS2probe	HEX-TCAGACACGCGGTCCGCTATAACGA-BHQ1	2278–2302

**Table 2:** Results obtained with CCHFV assays using HAZV hyperimmune sera.

Diagnostic Assay Incl. Antigen		ELISA			iIFA	Western Blot
		Vector-Best	IDVet Double Antigen	In-House	Euroimmun	In-House
		Inactivated whole virus, clade IV	Recombinant N protein, clade III	Recombinant N protein, clade V	Transfected cells with GPC and N protein, clade III	Recombinant N protein, clade V
**Serum**	Immunized calf	negative	negative	negative	weak positive	weak positive
	Immunized sheep	positive	negative	n/a *	weak positive	weak positive

* The post-immunization serum lead to high non-specific reactions in wells containing the antigen dilution buffer only.

## References

[1] Abudurexiti A., Adkins S., Alioto D., Alkhovsky S.V., Avšič-Županc T., Ballinger M.J., Bente D.A., Beer M., Bergeron E., Blair C.D. (2019). Taxonomy of the order Bunyavirales: Update 2019. Arch. Virol..

[2] Frias-Staheli N., Medina R.A., Bridgen A., Plyusnin A., Elliott R.M. (2011). Nairovirus Molecular Biology and Interaction with Host Cells. Bunyaviridae: Molecular and Cellular Biology.

[3] Clerx J.P.M., Casals J., Bishop D.H.L. (1981). Structural Characteristics of Nairoviruses (Genus Nairovirus, Bunyaviridae). J. Gen. Virol..

[4] Casals J., Tignor G.H. (1980). The Nairovirus Genus: Serological Relationships. Intervirology.

[5] Zeller H.G., Karabatsos N., Calisher C.H., Digoutte J.-P., Cropp C.B., Murphy F.A., Shope R.E. (1989). Electron microscopic and antigenic studies of uncharacterized viruses. II. Evidence suggesting the placement of viruses in the familyBunyaviridae. Arch. Virol..

[6] Bente D.A., Forrester N.L., Watts D.M., McAuley A.J., Whitehouse C.A., Bray M. (2013). Crimean-Congo hemorrhagic fever: History, epidemiology, pathogenesis, clinical syndrome and genetic diversity. Antivir. Res..

[7] Messina J.P., Pigott D.M., Golding N., Duda K.A., Brownstein J.S., Weiss D.J., Gibson H., Robinson T.P., Gilbert M., Wint G.R.W. (2015). The global distribution of Crimean-Congo hemorrhagic fever. Trans. R. Soc. Trop. Med. Hyg..

[8] Hoogstraal H. (1979). Review Article 1: The Epidemiology of Tick-Borne Crimean-Congo Hemorrhagic Fever in Asia, Europe, and Africa23. J. Med. Èntomol..

[9] Dowall S.D., Findlay-Wilson S., Rayner E., Pearson G., Pickersgill J., Rule A., Merredew N., Smith H., Chamberlain J., Hewson R. (2012). Hazara virus infection is lethal for adult type I interferon receptor-knockout mice and may act as a surrogate for infection with the human-pathogenic Crimean–Congo hemorrhagic fever virus. J. Gen. Virol..

[10] Begum F., Wisseman C.L., Casals J. (1970). Tick-borne viruses of West Pakistan. II. Hazara virus, a new agent isolated from Ixodes redikorzevi ticks from the Kaghan Valley, W. Pakistan. Am. J. Epidemiol..

[11] Begum F., Wisseman C.L., Traub R. (1970). Tick-borne viruses of west pakistan. Am. J. Epidemiol..

[12] Darwish M.A., Ghazi R., Amer T., Hoogstraal H., Roberts T.J. (1983). A sero-epidemiological survey for Bunyaviridae and certain other arboviruses in Pakistan. Trans. R. Soc. Trop. Med. Hyg..

[13] Shanmugam J., Smirnova S.E., Chumakov M.P. (1976). Presence of antibody to arboviruses of the Crimean Haemorrhagic Fever-Congo (CHF-Congo) group in human beings and domestic animals in India. Indian J. Med. Res..

[14] Subcommittee on Information Exchange (1970). Hazara (HAZ) strain. JC 280. Am. J. Trop. Med. Hyg..

[15] Marczinke B.I., Nichol S.T. (2002). Nairobi Sheep Disease Virus, an Important Tick-Borne Pathogen of Sheep and Goats in Africa, Is Also Present in Asia. Virology.

[16] Honig J.E., Osborne J.C., Nichol S.T. (2004). The high genetic variation of viruses of the genus Nairovirus reflects the diversity of their predominant tick hosts. Virology.

[17] Molinas A., Vikström E., Mirazimi A., Holm A., Loitto V.M., Magnusson K.-E. (2016). Protective role of host aquaporin 6 against Hazara virus, a model for Crimean–Congo hemorrhagic fever virus infection. FEMS Microbiol. Lett..

[18] Flusin O., Vigne S., Peyrefitte C.N., Bouloy M., Crance J.-M., Iseni F. (2011). Inhibition of Hazara nairovirus replication by small interfering RNAs and their combination with ribavirin. Virol. J..

[19] Molinas A., Turkina M.V., Magnusson K.-E., Mirazimi A., Vikström E. (2017). Perturbation of Wound Healing, Cytoskeletal Organization and Cellular Protein Networks during Hazara Virus Infection. Front. Cell Dev. Biol..

[20] Surtees R., Dowall S.D., Shaw A., Armstrong S., Hewson R., Carroll M.W., Mankouri J., Edwards T.A., Hiscox J.A., Barr J.N. (2016). Heat Shock Protein 70 Family Members Interact with Crimean-Congo Hemorrhagic Fever Virus and Hazara Virus Nucleocapsid Proteins and Perform a Functional Role in the Nairovirus Replication Cycle. J. Virol..

[21] Charlton F.W., Hover S., Fuller J., Hewson R., Fontana J., Barr J.N., Mankouri J. (2019). Cellular cholesterol abundance regulates potassium accumulation within endosomes and is an important determinant in bunyavirus entry. J. Biol. Chem..

[22] Matsumoto Y., Ohta K., Nishio M. (2018). Lethal infection of embryonated chicken eggs by Hazara virus, a model for Crimean-Congo hemorrhagic fever virus. Arch. Virol..

[23] Schuster I., Mertens M., Mrenoshki S., Staubach C., Mertens C., Brüning F., Wernike K., Hechinger S., Berxholi K., Mitrov D. (2016). Sheep and goats as indicator animals for the circulation of CCHFV in the environment. Exp. Appl. Acarol..

[24] Spengler J.R., Bergeron É., Rollin P.E. (2016). Seroepidemiological Studies of Crimean-Congo Hemorrhagic Fever Virus in Domestic and Wild Animals. PLoS Neglected Trop. Dis..

[25] Malik S., Diju I.U., Naz F. (2014). Crimean Congo hemorrhagic fever in Hazara division. J. Ayub Med. Coll. Abbottabad JAMC.

[26] Buckley S.M. (1974). Cross Plaque Neutralization Tests with Cloned Crimean Hemorrhagic Fever-Congo (CHF-C) and Hazara Viruses. Exp. Biol. Med..

[27] Gutjahr B., Keller M., Rissmann M., Von Arnim F., Jäckel S., Reiche S., Ulrich R., Groschup M.H., Eiden M. (2020). Two monoclonal antibodies against glycoprotein Gn protect mice from Rift Valley Fever challenge by cooperative effects. PLoS Negl. Trop. Dis..

[28] Mertens M., Vatansever Z., Mrenoshki S., Krstevski K., Stefanovska J., Djadjovski I., Cvetkovikj I., Farkas R., Schuster I., Donnet F. (2015). Circulation of Crimean-Congo Hemorrhagic Fever Virus in the Former Yugoslav Republic of Macedonia Revealed by Screening of Cattle Sera Using a Novel Enzyme-linked Immunosorbent Assay. PLoS Negl. Trop. Dis..

[29] David-West T. (1973). Dugbe virus: A new tick-borne arbovirus from Nigeria. Trans. R. Soc. Trop. Med. Hyg..

[30] Montgomery E. (1917). On a Tick-Borne Gastro-Enteritis of Sheep and Goats Occurring in British East Africa. J. Comp. Pathol. Ther..

[31] Smirnova S.E. (1979). A comparative study of the Crimean hemorrhagic fever-Congo group of viruses. Arch. Virol..

[32] Nuttall P.A. (2019). Tick saliva and its role in pathogen transmission. Wien. Klin. Wochenschr..

[33] Leech S. (2015). Investigation into the Vector Competence of Ixodes Ricinus Ticks to Hazara Virus and Crimean-Congo Haemorrhagic Fever Virus. Ph.D. Thesis.

[34] OIE (2019). Manual of Diagnostic Tests and Vaccines for Terrestrial Animals.

[35] Walker P.J., Widen S.G., Wood T.G., Guzman H., Tesh R.B., Vasilakis N. (2016). A Global Genomic Characterization of Nairoviruses Identifies Nine Discrete Genogroups with Distinctive Structural Characteristics and Host-Vector Associations. Am. J. Trop. Med. Hyg..

[36] Davies F., Casals J., Jesset D., Ochieng P. (1978). The serological relationships of Nairobi sheep disease virus. J. Comp. Pathol..

